# Differential Gene Expression Profile Induced by Valproic Acid (VPA) in Pediatric Epileptic Patients

**DOI:** 10.3390/genes9070328

**Published:** 2018-06-28

**Authors:** Esaú Floriano-Sánchez, Fernando Brindis, Daniel Ortega-Cuellar, Ivan Ignacio-Mejía, Elizabeth Moreno-Arriola, Pablo Romero-Morelos, Edgar Ceballos-Vasquez, María Guadalupe Córdova-Espinoza, Cindy Karel Arregoitia-Sarabia, Roberto Sandoval-Pacheco, Liliana Carmona-Aparicio, Noemí Cárdenas-Rodríguez

**Affiliations:** 1 Multidisciplinary Research Laboratory, Military Graduate School of Health, SEDENA, 11200 Mexico City, Mexico; florianoesa@hotmail.com (E.F.-S.); brindis77@unam.mx (F.B.); ivanignacio402@gmail.com (I.I.-M.); pablo.r.morelos@gmail.com (P.R.-M.); cevas0484@gmail.com (E.C.-V.); mixtlipp@yahoo.com.mx (M.G.C.-E.); 2 Laboratory of Experimental Nutrition, National Institute of Pediatrics, 04530 Mexico City, Mexico; dortegadan@gmail.com; 3 Genetics Unit of Nutrition, Institute of Biomedical Research UNAM—National Institute of Pediatrics, 04530 Mexico City, Mexico; elizamor86@hotmail.com; 4 Bacteriology Medical Laboratory, Microbiology Department, National School of Biological Sciences, National Polytechnic Institute, 11340 Mexico City, Mexico; 5 Laboratory of Neurosciences, National Institute of Pediatrics, 04530 Mexico City, Mexico; cindy_arregoitia@hotmail.com (C.K.A.-S.); c_apariccio@yahoo.com.mx (L.C.-A.); 6 Section of Research and Graduate Studies, National Polytechnic Institute, 11340 Mexico City, Mexico; 7 Military Hospital of Specialties of Women and Neonatology, Service of Emergency, SEDENA, 11200 Mexico City, Mexico; drsandovalpacheco@hotmail.com

**Keywords:** epilepsy, valproic acid, microarrays, transcriptome analysis

## Abstract

Epilepsy is a neuronal disease that affects up to 70 million people worldwide. The development of effective therapies to combat childhood epilepsy requires early biomarkers. Here, we performed a whole-genome microarray analysis in blood cells to identify genes differentially expressed between epileptic and epileptic valproic acid (VPA)-treated children versus normal children to obtain information about the gene expression to help us to understand genetic aspects of this disease. We found that the most significant differentially expressed genes were related to the transcriptional factor cAMP-response element binding protein (CREB) that is overexpressed in children with epilepsy compared with normal children, and 6 and 12 months of VPA treatment reversed several of these changes. Interestingly, leukocyte-associated immunoglobulin-like receptor 1 (LAIR1), a type I transmembrane glycoprotein that binds collagen proteins and contains CREB binding sites, was one of the more up-regulated genes in epileptic patients, and treatment with VPA strongly reversed its up-regulation. CREB up-regulates genes related to epilepsy; here, we suggest that LAIR1 could activate CREB, and together, they trigger epilepsy. After VPA treatment, LAIR1 repressed genes by disrupting the functional LAIR1–CREB complex, resulting in successful treatment. A functional microarray analysis offers new information that could open novel avenues of research in biomarker discovery, which may be useful for the early identification of children with a predisposition to epilepsy.

## 1. Introduction

According to the International League Against Epilepsy (ILAE), epilepsy is defined as a disease of the brain characterized by the occurrence of two unprovoked seizures more than 24 h apart, one unprovoked seizure and a probability of further seizures similar to the general recurrence risk (at least 60%) after two unprovoked seizures occurring over the next 10 years, or the diagnosis of an epilepsy syndrome [1]. This condition affects many people; in a recent study, it was reported that 70 million people have epilepsy worldwide, and approximately 90% of them are in developing regions, with a major prevalence in rural zones [2]. In addition, an estimated incidence has been reported of just over 100 patients per 1,000,000 inhabitants, with a prevalence of 3.7% in the population group aged 18–64 years, 1.6% in urban areas and 2.1–4.1% in rural children [3,4]. This condition has many risk factors, including family history, congenital, infections, trauma, neuronal diseases and malformations [2]. Epilepsy is characterized by many molecular mechanisms related to the immune system, such as deregulation of synaptic transmission, brain plasticity, apoptosis, neuroinflammation, oxidative stress and other functional alterations in the neuronal and neurovascular unit [5,6,7,8,9], resulting in gene expression alterations [10]. Antiepileptic drugs (AEDs) are used for epilepsy control, and valproic acid (VPA) is one of the more commonly used AEDs [11]. Several studies have demonstrated that AEDs (included VPA) may induce injury or show a neuroprotective effect in experimental models and humans [8,9,12].

Valproic acid is a branched short-chain fatty acid and is a widely used drug for the treatment of epilepsy, migraines and bipolar disorders [13]. In the treatment of epilepsy, VPA has several biochemical and molecular mechanisms: (a) it enhances inhibitory GABAergic activity and inhibits glutamatergic transmission [14,15]; (b) it modulates sodium and potassium channels [16,17]; (c) it modulates antioxidant defense and the production of oxidant metabolites [8]; (d) it modulates the activity and expression of protein kinases [18]; (e) it modulates neurogenesis, neuronal differentiation and neuronal survival [19,20]; and (f) it exerts effects on gene expression regulation by acting on transcription factors (mainly by regulating phosphorylation) and by acting as a histone deacetylase inhibitor [21,22].

We hypothesized that administration of VPA as an AED might alter the expression of some relevant genes related to neuroprotection and neurotoxicity pathways in patients with epilepsy. Here, we analyzed, for the first time, the gene expression profile of epileptic children as well as the molecular mechanism of VPA after different periods of administration in these patients by performing genome-wide array analysis to obtain novel transcript-based biomarkers that are predictive of epilepsy.

## 2. Material and Methods

### 2.1. Patients

All samples were collected from the Emergency Services, Military Hospital of Specialties of Women and Neonatology, Secretary; of National Defense (Secretaría de la Defensa Nacional, SEDENA) in Mexico City, Mexico. Two different groups of patients with available electronic health records were included in these experiments: (a) a group of 13 children (5 healthy children and 8 epileptic patients) were used for the microarray experiments, and (b) a confirmation group of 49 children (17 healthy children and 32 epileptic patients that followed 12 months of uninterrupted VPA monotherapy, including the patients in the microarray experiment) were used for the validation of some specific genes identified in the microarray analysis via real time-PCR (RT-PCR).

This study was approved by the Bioethics in Research Committee of the Military Hospital of Specialties of Women and Neonatology, SEDENA (Registration number 35, approval date 18 December 2015). The committee’s human experimentation guidelines were followed, and written informed consent was obtained from each patient.

The sample collection was conducted from December 2015 to March 2018 and each sample was considered according to the inclusion, exclusion and elimination criteria. The inclusion criteria were as follows: peripheral blood samples from newly admitted pediatric patients at the Military Hospital of Specialties of Women and Neonatology and the Central Military Hospital, SEDENA from 2016 to 2018; pediatric patients diagnosed with epilepsy or an epileptic syndrome; untreated pediatric patients for the first sampling and then 12 months of uninterrupted VPA treatment as the only AED (monotherapy). The exclusion criteria were as follows: pediatric patients with chronic diseases such as hematological, cardiac, hepatic, renal or thyroid disorders; pediatric patients with obesity; pediatric patients presenting infectious diseases or who had performed excessive physical exercise before sampling; pediatric patients taking drugs that interfere or alter the antioxidant or inflammatory state; pediatric patients taking an AED prior to the start of the study; and pediatric patients with a convulsive crisis with refractoriness of AED treatment. The elimination criteria were as follows: those cases of pediatric patients whose initial diagnosis of epilepsy was modified to another disease; pediatric patients who did not follow the therapeutic regimen during the study; and samples with low quality RNA. All recruited epileptic patients followed the inclusion, exclusion and elimination criteria, and none of the healthy children developed diseases or had a family history of epilepsy during the sampling. 

### 2.2. Blood Sampling and RNA Extraction

For the study with epileptic children, a 5 mL sample of peripheral venous blood was taken at the time of initial diagnosis (drug-free stage) and after VPA monotherapy (samples after 6 and 12 months of AED administration). A single blood sample was taken from the healthy children. The samples were collected in Vacutainer tubes containing EDTA (Becton, Dickinson and Company, Franklin Lakes, NJ, USA), and leucocytes were isolated. The isolation of RNA from the leucocytes was performed using Trizol reagent (TRI Reagent^®^ Solution, RNA/DNA/Protein Isolation Reagent, Invitrogen-Ambion, ThermoFisher Scientific, Waltham, MA, USA). Total cellular RNA was extracted according to the manufacturer’s protocol. RNA integrity, quality and quantification were assessed using QIAxcel and QIAxpert equipment (Qiagen, Germantown, MD, USA). All extracted RNA was preserved with nuclease inhibitor solution and stored at −80 °C.

### 2.3. Microarrays

Total RNA samples from five control children and eight epileptic children were analyzed using a microarray according to the manufacturer’s protocol (Two-Color Microarray-Based Gene Expression Analysis/Low Input Quick Amp Labeling; Agilent Technologies, Santa Clara, CA, USA). Briefly, 75 ng of total RNA was converted to cDNA, followed by in vitro transcription and incorporation of Cyanine 3-CTP into the nascent complementary RNA (cRNA), followed by fragmentation and hybridization to Agilent SurePrint Human GE 8 × 60 K Microarrays (Agilent Technologies) for 17 h at 65 °C. The quality control parameters used for cRNA labeling and hybridization were specified by the manufacturers. The microarrays were scanned using a NimbleGen microarray scanner (Roche, Basel, Switzerland), and the signal intensities in the TIFF images were calculated using Feature Extraction software (FE, version 12.0; Agilent Technologies). The microarray data were analyzed, and the associated biological pathways were determined using GeneSpring GX 13.0 software (Agilent Technologies). Differentially expressed genes were selected with a fold change >2.0 and *p* < 0.05. The Benjamini–Hochberg algorithm was used to compute false discovery rates [23]. The classification of the identified pathways was based on the Kyoto Encyclopedia of Genes and Genomes pathway database (KEGG Pathway Maps). The gene ontology analysis for down- and up-regulated genes was submitted to the bioinformatics and functional annotation tool of the Database for Annotation, Visualization and Integrated Discovery (DAVID Bioinformatics Resources) v. 6.8 of the NIAID (National Institute of Allergy and Infectious Disease), NIH (National Institutes of Health).

### 2.4. Gene Expression Validation

To confirm the microarray analysis results, RT-PCR validation was performed for the most representative dysregulated genes from the total sample population (which included 17 healthy control children and 32 children with epilepsy). Reverse transcription was performed using a One-Step qRT-PCR KAPA SYBR FAST^®^ Kit (Kapa Biosystems, Sigma-Aldrich, St. Louis, MO, USA) according to the manufacturer´s protocol, with a Rotor-Gene Q (Qiagen) and specific gene primers (provided upon request). To determine the relative gene expression levels, the the number of cycles in which the fluorescence intensity increases above the baseline fluorescence of the sample (CPs) of endogenous candidates genes (glyceraldehyde-3-phosphate dehydrogenase (*GAPDH)*, β-2-microglobulin (*B2M)* and actin-β (*BACT)*) and each of the analyzed genes were exported from Rotor-Gene Q v.2.3.1 software (Qiagen) to calculate the efficiencies using the *REST©* statistical model [24,25], and the data were plotted by constructing a linear regression comparing the logarithmic concentration (total RNA) against the CP. To correlate the candidate endogenous genes and determine the more stably expressed genes, BestKeeper software was used by exporting the CP values from the Rotor-Gene Q software using the Excel tool to show the melting temperature (Tm) characteristics of each amplified product. The expression levels of the housekeeping genes were analyzed using the BestKeeper statistical model, which analyzed the CP values via Pearson correlation [26].

### 2.5. Statistical Analysis

All statistical analyses were performed using GraphPad Prism version 6.0 software (La Jolla, CA, USA) and XLSTAT for Excel 2018 (Addinsoft, NY, USA). The data are expressed as the mean ± standard deviation (SD). The Kolmogorov–Smirnov normality test was performed based on the null hypothesis that the data was normally distributed. Data from the absolute quantification of all the samples were normalized with housekeeping genes and were analyzed using the Student’s *t*-test. Differences between groups were tested using analysis of variance (ANOVA) with Bonferroni post hoc analysis.

## 3. Results

### 3.1. Characteristics of the Patients Included in the Microarray Study

The random sample for the microarray study consisted of 13 children, distributed with respect to weight and sex. Overall, 13 patients participated, with five children in the healthy group and eight epileptic children in the drug-free and VPA monotherapy group, with a range of ages (months of age, mean ± SD) between 36.6 ± 13, and 58.3 ± 55.2 for healthy and epileptic children, respectively. Of the healthy children, 40% were female and 60% were male, and of the epileptic children, 25% were female and 75% were male. The characteristics of the patients with epilepsy used in the microarray study are presented in Table 1. In terms of family history of epilepsy, 3 (37.5%) had relatives with epilepsy, 3 (37.5%) had a perinatal history, 2 (25%) presented with other diseases, 6 (75%) showed generalized epilepsy with idiopathic etiology, 6 (75%) presented abnormal findings in the image studies, and 6 (75%) had completely controlled epilepsy. 

The general characteristics of all the 32 patients treated with VPA monotherapy is as follows: the mean actual age was 6.4 ± 4.1 years (range of 2–15 years), and the age at the beginning of VPA treatment was 4.2 ± 4.1 years (range of 0–12 years) and the mean body mass index (kg/m^2^) was 16.9 ± 2.7. Among the patients, 9 were female and 23 were male. In terms of family history of epilepsy; 6 patients had relatives with epilepsy and 17 patients had generalized epilepsy and idiopathic etiology and 4 patients had comorbidities; 12 patients had abnormal brain findings on imaging studies; the mean serum level of VPA (μg/mL) was 28.9 ± 16.9 and the mean weight-based divided dose of VPA (mg/kg) was 24.04 ± 9.34.

### 3.2. Gene Expression Profiling in Peripheral Blood Cells

**Figure 1:**
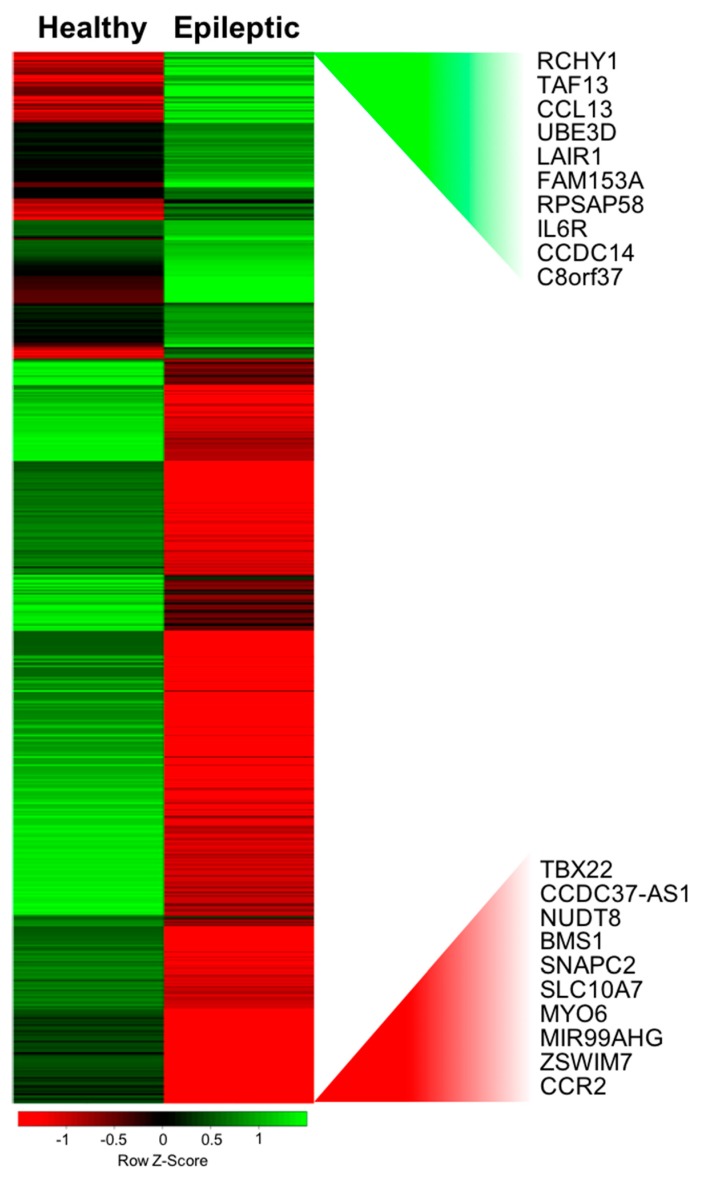
Gene expression changes triggered by epileptic disease. Microarray heat map shown genes that were differentially expressed and unchanged during epileptic episodes without treatment. A color code scale was used to show gene expression differences in logarithmic fold change units between the groups (red represents lower expression; green represents higher expression).

Of the 50,378 probes tested in our microarray analysis, 21,056 probes remained after background correction and were used for further analysis. In total, 978 unique genes were found to be significantly differentially expressed between normal and epileptic children before treatment. There were 341 unidentified genes in a microarray data set and these were not considered in our analysis. From the remaining 637 known coding genes, 451 were down-regulated, and 186 were up-regulated in epileptic children compared to normal children (Table S1, Supplementary Materials). The results from the array concerning the 637 genes differentially expressed between both conditions are depicted in a heat map (see Figure 1).

### 3.3. Functional Analysis

To study the functions of the differentially expressed genes, DAVID Bioinformatics Resources were applied to known gene ontology pathways. The 637 known genes with significantly different expression levels between epileptic patients before treatment and normal children were classified into several biological processes according to their function. The biological processes showing the highest number of differentially expressed genes were related to transcription/translation machinery (*n* = 28), Poly(A) RNA binding (*n* = 51), a cytokine-mediated signaling pathway (*n* = 11), the immune system (*n* = 19), cytokine-cytokine receptor interactions (*n* = 14), DNA binding (*n* = 67), protein binding (*n* = 254), and positive regulation of smooth muscle cell proliferation (*n* = 5), among other functions. The functional annotation clustering of genes differentially expressed are shown in Table 2. Notably, most of the genes involved in these pathways were up-regulated, and they had not previously been associated with epileptic children. 

Additionally, we applied bioinformatics analyses to detect any enrichment of transcriptional factors. We found a cluster of 349 of the 637 genes with significant expression (54.8%) with CRE motifs (TGACGTCA), whose transcription is controlled by the cAMP-response element binding protein (CREB) transcription factor in the transcriptome of epileptic children before treatment (in comparison with healthy children). Of these 349 CREB genes, 106 were up-regulated (30.4%), and 243 were down-regulated (69.6%) (Table S2, Supplementary Materials). The results for the ten genes related to CREB that displayed the greatest changes are shown in Table 3. 

**Figure 2:**
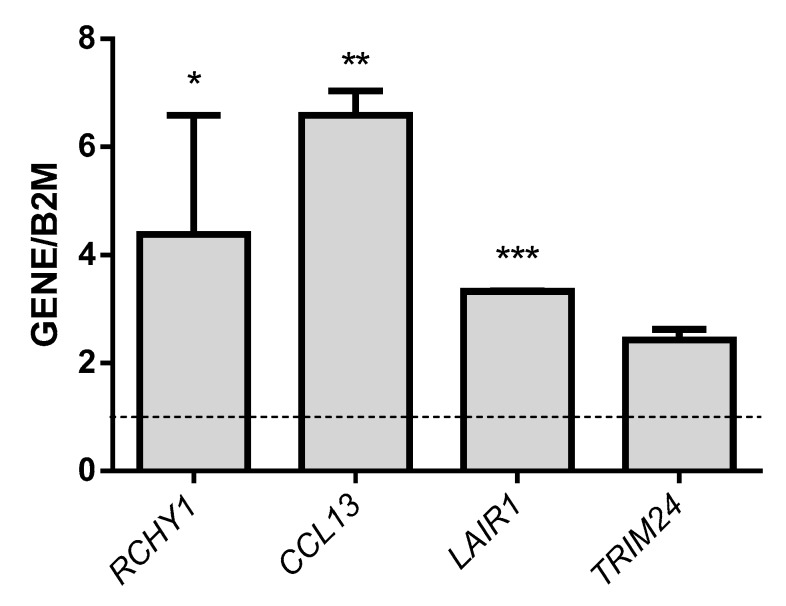
Levels of normalized mRNA of the up-regulated genes *RCHY1*, *CCL13*, *LAIR1* and *TRIM24* in pediatric patients with epilepsy. The gene levels were normalized to the level of the housekeeping gene *B2M* and are expressed per 20 ng of total RNA. The values represent the means with standard deviations (SD) of the expression levels of each gene. We found significant differences between all genes. * *p* < 0.05 vs. the gene expression of *CCL13*, *LAIR1*, and *TRIM24*; ** *p* < 0.05 vs. the gene expression of *LAIR1* and *TRIM24*; and *** *p* < 0.05 vs. the gene expression of *TRIM24*.

**Figure 3:**
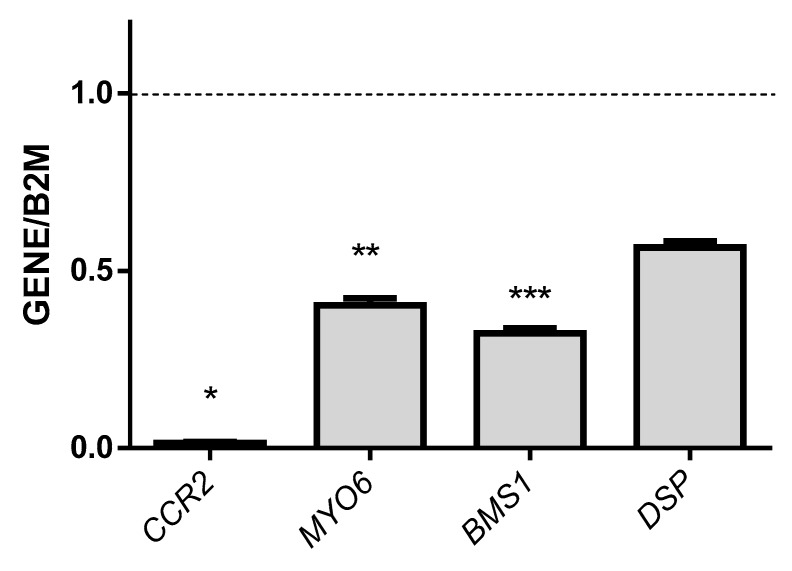
Levels of normalized mRNA of the down-regulated genes *CCR2*, *MYO6*, *BMS1* and *DSP* in pediatric patients with epilepsy. The gene levels were normalized to the level of the housekeeping gene *B2M* and are expressed per 20 ng of total RNA. The values represent the means with standard deviations (SD) of the expression levels of each gene. We found significant differences between all genes. * *p* < 0.05 vs. the gene expression of *MYO6*, *BMS1*, and *DSP*; ** *p* < 0.05 vs. the gene expression of *BMS1* and *DSP*; and *** *p* < 0.05 vs. the gene expression of *DSP*.

Real time-PCR was used to validate the expression of selected genes related to the CREB transcription factor: *RCHY1*, *CCL13*, *LAIR1 (*leukocyte-associated immunoglobulin-like receptor 1), *TRIM24*, *CCR2*, *MYO6*, *BMS1* and *DSP*. The results showed that *RCHY1*, *CCL13*, *LAIR1* and *TRIM24* were significantly up-regulated genes and *CCR2*, *MYO6*, *BMS1* and *DSP* were significantly down-regulated genes in pediatric patients with epilepsy (see Figure 2 and Figure 3).

### 3.4. Association Studies of Gene Expression in Epileptic Patients Undergoing Valproic Acid Monotherapy

**Figure 4:**
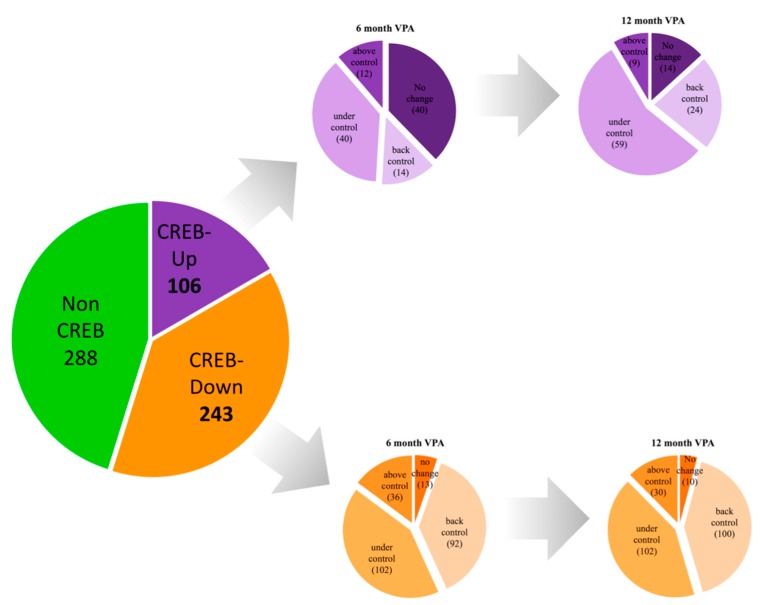
The image shows the profile of changes provoked by the administration of VPA (6 and 12 months) to pediatric patients with established epilepsy on the genes that depend on the transcriptional factor cAMP-response element binding protein (CREB).

**Figure 5:**
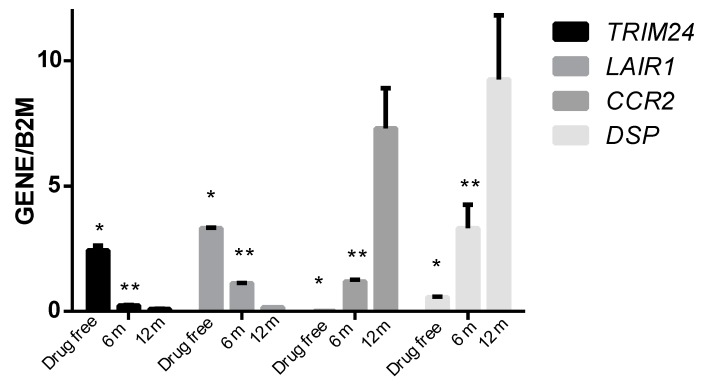
Normalized mRNA levels of the genes *TRIM24*, *LAIR1*, *CCR2* and *DSP* in the drug-free stage and after 6 and 12 months of VPA treatment in pediatric patients with epilepsy. The gene levels were normalized to the level of the housekeeping gene *B2M* and are expressed per 20 ng of total RNA. The values represent the means with standard deviations (SD) of the expression levels of each gene. * *p* < 0.05 vs. the level of gene expression after 6 and 12 months of VPA treatment, and ** *p* < 0.05 vs. the levels of gene expression after 12 months of VPA treatment.

To investigate the effect of VPA monotherapy on the change in the expression of several mRNAs in epileptic patients, we selected the CREB genes to measure again after 6 and 12 months of VPA monotherapy. As shown in Figure 4, 106 genes of the 637 genes with significant expression (16.6%) were up-regulated by epilepsy in epileptic children before treatment. The expression of 14 of them returned to healthy levels after six months of VPA treatment, whereas the expression level of 40 genes began to decrease after six months and continued decreasing after 12 months of treatment with VPA. Consistent with the progression of the VPA treatment time and the improvement in epileptic episodes at 12 months, the gene expression levels of 92 of the 106 transcripts (86.8%) were changed. Interestingly, we found that six months of monotherapy were unable to change the expression of 40 genes, while after 12 months of monotherapy, only 14 remained unchanged. Among the genes that were highly expressed in the epileptic group, we found *RCHY1*, *CCL13*, *LAIR-1*, *RPSAP58*, *IL6R*, *CCDC14*, *AFAP1L2*, *TRIM24*, *CSMD1*, *ZNF704*, *CDK5RAP3*, *FRAS1*, *SKA1*, *CYS1*, *IL5*, *LHFP* and *CPB1*. Interestingly, VPA treatment reduced their expression, suggesting that these genes promote epilepsy, since this condition was also diminished as the treatment period progressed. Specifically, *RCHY1*, *CCL13*, *LAIR-1*, *RPSAP58* and *TRIM24* were down-regulated after six months of VPA treatment. In the validation experiment, we observed that the expression levels of *TRIM24* and *LAIR1* were significantly decreased after six months of VPA treatment, whereas the expression levels of *CCR2* and *DSP* were significantly increased after six months of VPA treatment compared with the drug-free stage (see Figure 5). *TRIM24* gene expression decreased by 90.5% and 96% during 6 and 12 months of monotherapy, respectively, and *LAIR1* gene expression decreased by 66.1% and 95% during 6 and 12 months of monotherapy, respectively, compared with the drug-free stage. Between 6 and 12 months of VPA treatment, the gene expression of *TRIM24* and *LAIR1* decreased by 57.8% and 85.4%, respectively. *CCR2* gene expression increased by more than 1000% between 6 and 12 months of monotherapy, and *DSP* gene expression increased by more than 100% during the same time period compared with the drug-free stage. Between 6 and 12 months of VPA treatment, the gene expression of both *CCR2* and *DSP* increased by more than 100%.

In addition to the genes that increased during epilepsy and without treatment, we found that the expression of 243 genes of the 637 genes with significant expression in epileptic children before of treatment (38.1%) was decreased. Of these 243 genes, after treatment with VPA for 6 months, 13 genes were unchanged, 92 were expressed at healthy levels, the expression of 102 decreased more than expected, and 36 had increased expression levels. After 12 months of VPA treatment, only 10 of the 243 (4%) genes did not have changed expression levels (see Figure 4 and Table S3, Supplementary Materials). These results suggest that many of the benefits of VPA treatment of epilepsy may be attributed to the effect on the transcription of several genes.

## 4. Discussion

In the present study, we explored the transcriptome of leucocytes during epilepsy in pediatric patients before and after different lengths of treatment with VPA monotherapy. Microarray studies in pediatric patients with epilepsy indicated that short and long-term (6 and 12 months) exposure to the well-established therapeutic drug VPA induces altered mRNA levels of a large number of genes, and importantly, reverting most of the genes altered by the disease to a healthy level.

**Figure 6:**
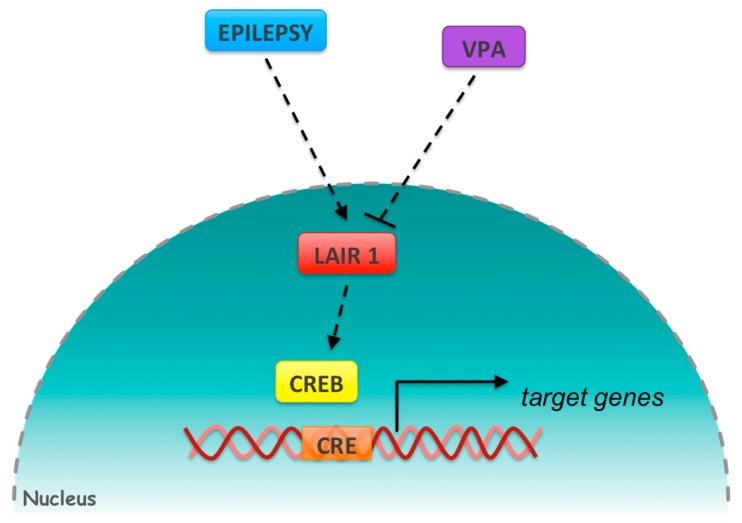
Image depicting a possible model of action for VPA in generalized epilepsy on gene expression through the LAIR1-CREB axis.

Potential mediators for the positive regulation of epileptic gene transcription include CREB [27], which is a well-known master regulator of metabolic pathways. CREB is an attractive candidate because it was recently identified as a key regulator of a pathway that involves LAIR-1 [28] (Figure 6). 

The *LAIR-1* gene encodes a transmembrane protein that inhibits the receptor C1q, the first complement component, and therefore, *LAIR-1* prohibits the signal transduction associated with the production of pro-inflammatory cytokines [29,30]. Other studies have shown that in the brain, C1q promotes synaptic pruning, preventing enhanced excitatory synaptic connectivity and epileptiform activity [31]. In fact, mice deficient in C1q have a spontaneous epileptogenic condition due to the failure of synaptic pruning, suggesting that the failure of the key factor C1q may lead to epileptogenesis [32]. Based on these findings, it is plausible to hypothesize that overexpressed *LAIR-1* could inhibit the activity of C1q, consequently leading to the generation of seizures in epilepsy. Furthermore, it has recently been demonstrated that *LAIR-1* triggers a CREB-dependent signaling pathway that leads to myeloid leukemia development [28]. Since the *LAIR-1* promoter contains a CRE sequence, it is possible that CREB may increase the transcription of *LAIR-1*, generating a transcriptional regulatory loop to establish a CREB-driven transcriptional program that conceivably may trigger epilepsy. Strikingly, the increased expression of *LAIR-1* in patients with epilepsy was strongly diminished after 6 and 12 months of VPA treatment, and this observation could be correlated with decreased brain seizure activity. 

Another gene overexpressed in children with epilepsy was *IL6R*, whose promoter region contains a CRE sequence. It has been reported that IL6R and its ligand, IL6, have both detrimental and beneficial effects on the nervous system. Specifically, IL-6 and IL6R are closely associated with neurological excitability [33]; thus, it has been proposed that this pathway may be involved in epilepsy. The *TRIM24* gene encodes an E3 ubiquitin ligase [34], and defects in the ubiquitin proteasome system have long been implicated in the pathogenesis of neurodegenerative disorders. Specifically, TRIM2, which has been shown to be highly expressed in the nervous system, binds to the neurofilament light subunit (NF-L), regulating its ubiquitination. If it is deregulated, it triggers neurodegeneration [35]. Given its related function, *TRIM24* might play a similar role in the development of epilepsy. As with *LAIR-1*, we demonstrated that VPA treatment also dramatically down-regulates its expression throughout the entire 12 months of VPA treatment.

The CUB structural domaine (for complement C1r/C1s, Uegf and Bmp1) and sushi multiple domains 1 gene, *CSMD1*, which is highly expressed in epithelial tissues and the central nervous system, plays a role as an important regulator of complement initiation and inflammation. Patients who carry a deletion of this gene show language delay, learning difficulties and epilepsy [36]. Spindle and kinetochore associated complex subunit 1 (SKA1), has been suggested as being involved in human brain development [37]; however, its participation in epilepsy has not been studied. 

Our data showed that changes in the expression of the above mentioned genes were diminished after VPA treatment. The gene *C21orf131* is known to be highly expressed in the human brain, but its specific functions are not clear, nor is it known whether it has lower expression in patients with epilepsy. This study is the first report showing neurodevelopment problems related to the *C21orf131* gene. We suggest that it is necessary to investigate the role of this gene to understand the development of human epilepsy. 

In addition to the increased transcription of CRE-regulated promoters during epilepsy in pediatric patients and the decrease in their transcription through VPA monotherapy treatment, we also observed a set of genes containing CRE motifs with decreased expression in epileptic patients. After VPA treatment, this group of genes showed a tendency to increase their expression levels.

Regarding mRNA with decreased expression levels during epileptic episodes in children, most had reverted levels of expression after 6 or 12 months of treatment with VPA; those genes were *CCR2*, *ZSWIM7*, *MYO6*, *SNAPC2*, *BMS1*, *TBX22*, *MKLN1-AS*, *EFTUD2*, *IDH3B*, and *DSP*. 

Elevated levels of the chemokine C-C motif ligand 2 receptor CCR2 have been reported in experimental seizures, and the neuronal localization of CCR2 has been mapped [38,39]. However, its participation in relieving episodes of seizures due to VPA treatment has not been widely evaluated, and we demonstrated that after 6 months of monotherapy with VPA, the large reduction in *CCR2* transcript levels was ameliorated as the treatment progressed, thus possibly contributing to the reduction in seizures. MYO6, an unconventional myosin, has been previously associated with deafness [40]. BMS1 is a ribosome biogenesis factor already reported to be involved in epilepsy. Its reversion to control levels due to VPA and its participation in the development of convulsive episodes has not been studied. 

The decrease of the *ZSWIM7*, *MYO6*, *SNAPC2*, *BMS1*, *TBX22*, *MKLN1-AS*, *EFTUD2*, *IDH3B*, and *DSP* transcripts had not been previously associated with epileptic episodes in pediatric patients, and even less is known about their re-establishment by monotherapeutic treatment with VPA. We confirmed that the gene expression of *DSP*, similar to *CCR2*, increased dramatically throughout the 12 months of VPA monotherapy. This generates new horizons in the study of signaling pathways for the treatment and prevention of epileptogenesis. Diminished expression of this group of genes could be mediated by ICER, an inducible cAMP early repressor, which is part of the CRE binding protein family that shares the ability to also bind to the highly conserved DNA 8-mer (TGACGTCA) sequence, a CRE motif [41,42]. In laboratory models, following kainic acid-induced or electroconvulsive seizures, there is an increase in the level of ICER [42], which could explain the blocking of the expression of a specific group of genes that contain the CRE motif and could consequently explain why treatment with VPA eliminates this blockage and re-establishes the expression of most genes. It is relevant to mention that these alterations in the expression of genes are from patients with generalized epilepsy with principally idiopathic etiology. Other studies will be focused on determining the effects of VPA in other kinds of seizures with other originating causes in epileptic children. 

## 5. Conclusions

Functional microarray analysis suggests a possible therapeutic gene bank that may be a useful approach for early identification of children with predispositions to epilepsy. Moreover, our results showed that VPA differentially regulates the gene expression profile in epileptic children during the 6 and 12 months of treatment, principally in one group of genes related with the CREB transcription factor, suggesting the LAIR1-CREB axis as a possible action mechanism of this AED in generalized epilepsy.

## Figures and Tables

**Table 1:** Clinicopathological characterization of the patients included in the microarray study. VPA: Valproic Acid.

Patient	Age at the Beginning of Treatment (Months)	Family History of Epilepsy	Perinatal History	Other Diseases	Type of Convulsion	Etiology of Epilepsy	Findings in the Image Studies	Mean Daily Convulsions before Treatment	Mean Daily Convulsions after Treatment	VPA Dose (mg/kg of Body Weight)
1	12	None	Prematurity	None	Generalized	Idiopathic	Left cortical atrophy	4	0	30
2	144	Mother diagnosed with epilepsy	Threatened abortion	None	Generalized	Idiopathic	None	1	0	48
3	84	Aunt diagnosed with epilepsy	None	None	Generalized	Idiopathic	Abnormal paroxysm	6	0	25
4	132	No	None	Asthma	Generalized	Idiopathic	Irritative cortical activity	1	0	10
5	48	Brother diagnosed with epilepsy	None	None	Generalized	Idiopathic	Ventricular asymmetry	18	0	20
6	12	None	Prematurity	Bronchopulmonary dysplasia	Generalized	Symptomatic	Epileptiform activity in the left parietal region	1	1	20
7	10	None	None	None	Generalized	Idiopathic	Ventricular asymmetry with increase in left occipital volume	5	0	25
8	24	None	None	None	Generalized	Symptomatic	None	3	1	38.9

**Table 2:** Functional annotation clustering of genes differentially expressed in epileptic children.

Pathway	*n*	*p*-Value	Fold Enrichment	Benjamini False Discovery Rate
Translation	28	6.12 × 10^−10^	4.2236	2.18 × 10^−7^
Poly(A) RNA binding	51	3.12 × 10^−4^	1.6834	0.0866
Coiled-coil	104	0.0016	1.3327	0.1019
Cytokine-mediated signaling pathway	11	0.0023	3.2046	0.2925
Alternative splicing	304	0.0032	1.1171	0.1449
Receptor binding	20	0.0032	2.1113	0.3725
Cytokine-cytokine receptor interaction	14	0.0075	2.2984	0.1655
Immune response	19	0.0288	1.7223	0.9340
DNA-binding	67	0.0317	1.2715	0.6044
Jak-STAT signaling pathway	9	0.0372	2.3437	0.4948
Regulation of immune response	10	0.0446	2.1440	0.9505
Protein binding	254	0.0494	1.0774	0.8959
Phosphoprotein	230	0.0640	1.0852	0.7827
Positive regulation of smooth muscle cell proliferation	5	0.0716	3.1803	0.9818
Non-syndromic deafness	6	0.0730	2.6831	0.806
Metal-binding	107	0.0844	1.1437	0.799
Zinc ion binding	40	0.0919	1.2751	0.9389
Adaptive immune response	8	0.0938	2.0629	0.9861

**Table 3:** Most representative deregulated cAMP-response element-genes (CRE-genes) in pediatric patients with epilepsy in comparison with healthy children.

**Up-Regulated CRE-Genes**		
**Official Symbol**	**Official Full Name**	**Fold Change**
RCHY1	Ring finger and CHY zinc finger domain containing 1	193.7847
CCL13	C-C motif chemokine ligand 13	133.6095
LAIR1	Leukocyte associated immunoglobulin-like receptor 1	80.0391
RPSAP58	Ribosomal protein SA pseudogene 58	62.9540
IL6R	Interleukin 6 receptor	60.4950
CCDC14	Coiled-coil domain containing 14	49.6876
C21orf131	Long intergenic non-protein coding RNA 320	42.6516
AFAP1L2	Actin filament associated protein 1-like 2	36.8321
TRIM24	Tripartite motif containing 24	14.5041
CSMD1	CUB and Sushi multiple domains 1	13.5815
**Down-Regulated CRE-Genes**		
**Official Symbol**	**Official Full Name**	**Fold Change**
CCR2	C-C motif chemokine receptor 2	−190.5848
ZSWIM7	Zinc finger SWIM-type containing 7	−167.3231
MYO6	Myosin VI	−148.6852
SNAPC2	Small nuclear RNA activating complex polypeptide 2	−118.0506
BMS1	Ribosome biogenesis factor	−108.2960
TBX22	T-box 22	−78.6303
MKLN1-AS	MKLN1 antisense RNA	−69.2440
EFTUD2	Elongation factor Tu GTP binding domain containing 2	−64.2970
IDH3B	Isocitrate dehydrogenase 3 (NAD(+)) beta	−61.9831
DSP	Desmoplakin	−54.6393

## Supplementary Materials

The following are available online at http://www.mdpi.com/2073-4425/9/7/328/s1, Table S1: Complete list of differentially expressed genes between normal and epileptic children, identified by microarray analysis, Table S2: List of differentially expressed genes between normal and epileptic children with significantly enriched CRE motif, Table S3: List of differentially expressed genes with CRE motif in epileptic children and the effect of VPA administration. Orange: up-regulated, Purple: down-regulated.
