# Supplementary material for: Differential Gene Expression Profile Induced by Valproic Acid (VPA) in Pediatric Epileptic Patients
Source: Genes (Basel). 2018 Jun 28;9(7):328. doi: 10.3390/genes9070328 (PMC6070821; doi:10.3390/genes9070328)
Supplement: Supplementary file 1 [file genes-09-00328-s001.zip › genes-308394-supplementary.docx]

**Table S1.** Complete list of differentially expressed genes between normal and epileptic children, identified by microarray analysis.

| **GENE** | **Fold change in epileptic patients before treatment** |
| --- | --- |
| CCR2 | -190.58481 |
| ZSWIM7 | -167.32315 |
| MIR99AHG | -150.31482 |
| MYO6 | -148.68521 |
| SLC10A7 | -144.44907 |
| SNAPC2 | -118.050606 |
| BMS1 | -108.29604 |
| NUDT8 | -107.738846 |
| CCDC37-AS1 | -81.76495 |
| TBX22 | -78.630394 |
| MKLN1-AS | -69.24401 |
| LINC00158 | -68.86511 |
| USP17L2 | -64.66983 |
| EFTUD2 | -64.297066 |
| IDH3B | -61.983143 |
| DSP | -54.639378 |
| FOSL1 | -53.243614 |
| ZNF676 | -47.39888 |
| PRR19 | -46.496128 |
| UXT | -45.996002 |
| WHAMMP3 | -39.76804 |
| MYO7A | -37.412678 |
| COX6B2 | -36.6709 |
| ZNF713 | -36.121647 |
| LOC101929261 | -35.51971 |
| CAMK2B | -32.3308 |
| CDK11A | -31.870256 |
| HTR1F | -30.895006 |
| HYAL4 | -28.413912 |
| GPATCH2 | -28.376295 |
| TFF2 | -28.258219 |
| FAM118A | -28.252895 |
| BTAF1 | -27.195265 |
| ITFG1-AS1 | -26.28395 |
| AQP7P3 | -25.705 |
| CRYAA | -24.553106 |
| ADAMTSL4-AS1 | -23.984385 |
| TNRC18P1 | -23.906996 |
| MAP3K2 | -23.41734 |
| TRIM41 | -23.271374 |
| TBL2 | -22.315361 |
| VWDE | -21.8643 |
| KIAA1875 | -21.343864 |
| ZCCHC2 | -20.993662 |
| GOLGA2 | -20.509306 |
| OXLD1 | -19.668362 |
| TBXA2R | -18.730059 |
| ZNF385C | -17.51781 |
| ALOX15 | -17.440674 |
| FUT6 | -17.073818 |
| HDGFRP2 | -16.983545 |
| RPL21 | -16.737719 |
| MTRNR2L6 | -16.55654 |
| TTC28 | -16.516504 |
| POLR2J2 | -16.35658 |
| LINC00963 | -16.048397 |
| LINC00452 | -15.639537 |
| AS3MT | -15.374013 |
| CCDC159 | -15.312297 |
| SNORA15 | -15.257609 |
| GPR155 | -15.160358 |
| DHRS12 | -14.318231 |
| GARNL3 | -14.272562 |
| PCGEM1 | -14.019029 |
| NRG3 | -13.746782 |
| MAST4 | -13.602137 |
| ZNF582-AS1 | -13.512826 |
| NABP1 | -13.428069 |
| RNU4ATAC | -13.38707 |
| NR2F6 | -13.340603 |
| ZNF581 | -13.262683 |
| CTAGE7P | -13.1600685 |
| NMNAT1 | -12.252648 |
| GBP6 | -11.697837 |
| MIPEPP3 | -11.252195 |
| LOC284454 | -11.1111965 |
| CHPF | -10.699044 |
| LETM1 | -10.485501 |
| INPP4B | -10.285258 |
| SLC25A16 | -10.230792 |
| CEP70 | -9.674362 |
| RN7SL1 | -9.543825 |
| FAM74A4 | -9.512757 |
| OGFRL1 | -9.503074 |
| ZNF816 | -9.4184885 |
| TMEM191B | -9.3391075 |
| TMEM138 | -9.259202 |
| DPYSL5 | -9.110845 |
| FOXD1 | -9.050619 |
| MGA | -8.93599 |
| BTN3A1 | -8.81694 |
| TRIQK | -8.74564 |
| SBDS | -8.705719 |
| LURAP1L-AS1 | -8.602464 |
| ZNF385B | -8.541545 |
| PKD2L2 | -8.503096 |
| CYP2W1 | -8.469664 |
| MTRNR2L1 | -8.337105 |
| MTRNR2L2 | -8.268313 |
| LINC00608 | -8.24484 |
| LINC00399 | -8.143101 |
| CBR1 | -7.8004494 |
| PPP1R17 | -7.793646 |
| SLC25A29 | -7.74265 |
| RBM10 | -7.7352743 |
| MIR600HG | -7.7316604 |
| MTRNR2L2 | -7.634083 |
| TRNT1 | -7.4508705 |
| GREB1 | -7.3220215 |
| SLC16A12 | -7.203574 |
| IFNA14 | -7.187003 |
| HIST1H4H | -7.1246715 |
| MUC22 | -7.05274 |
| PRTFDC1 | -7.0514326 |
| RPS2 | -6.9343185 |
| MYCNUT | -6.926281 |
| CSTF2 | -6.9165263 |
| CHAT | -6.863287 |
| NRXN1 | -6.8505344 |
| DNM3 | -6.7793155 |
| CCDC22 | -6.7769365 |
| BTBD19 | -6.714665 |
| PDE4DIP | -6.6277466 |
| ATP6V1G3 | -6.597734 |
| FAM200B | -6.587315 |
| C14orf1 | -6.5065913 |
| RPS15 | -6.458646 |
| GUCY1B3 | -6.4555683 |
| TNRC18 | -6.39616 |
| LINC01405 | -6.3276873 |
| NKX3-1 | -6.2034426 |
| RPL9 | -6.1629453 |
| HAUS1 | -5.985859 |
| LOC100130950 | -5.975011 |
| ARAFP2 | -5.973538 |
| RPS2 | -5.944819 |
| AGPAT9 | -5.9159155 |
| HSCB | -5.869288 |
| KLF12 | -5.8542767 |
| TMEM8C | -5.836533 |
| PDCD4-AS1 | -5.791649 |
| CXADR | -5.7766285 |
| LINC01193 | -5.653984 |
| TRO | -5.499091 |
| RPL13 | -5.4776607 |
| LOC100506679 | -5.449681 |
| DUSP15 | -5.4436827 |
| PAXIP1 | -5.4378057 |
| HLA-G | -5.428023 |
| CD72 | -5.3975916 |
| ZNF593 | -5.3650537 |
| TMEM178B | -5.28342 |
| DOCK9 | -5.263303 |
| ZNF2 | -5.2576323 |
| OR1M1 | -5.22997 |
| SLC35E4 | -5.1544924 |
| HLA-A | -5.1290436 |
| MCM3AP-AS1 | -5.1062274 |
| TTPAL | -4.9871063 |
| WIPF1 | -4.9532194 |
| RPS11 | -4.910102 |
| LINC01001 | -4.8545 |
| ALPP | -4.8264446 |
| LMO7 | -4.807182 |
| BOC | -4.759694 |
| LOC102725254 | -4.729466 |
| LOC101929011 | -4.678003 |
| DUOX2 | -4.6750593 |
| ACTB | -4.635732 |
| SMAD3 | -4.593347 |
| XRCC3 | -4.543103 |
| CCDC9 | -4.476432 |
| NFYC | -4.451852 |
| PIEZO1 | -4.375065 |
| FTSJ2 | -4.3666067 |
| C10orf105 | -4.3469048 |
| GLUD1P3 | -4.252379 |
| HLA-B | -4.244339 |
| FAM166B | -4.237783 |
| ATP1A4 | -4.2278624 |
| MYO1C | -4.179342 |
| HLA-C | -4.1200385 |
| ATP8B5P | -4.1134977 |
| RPL21 | -4.0791345 |
| CASC5 | -4.0748143 |
| LOC440600 | -4.0355477 |
| MORN4 | -3.9924686 |
| PGLYRP1 | -3.9661927 |
| ACBD7 | -3.9648783 |
| ADAMTS7 | -3.9534247 |
| HOXA6 | -3.9459076 |
| EWSAT1 | -3.9178066 |
| ATP6 | -3.886592 |
| ATP2B3 | -3.856911 |
| RPL5 | -3.8556387 |
| EEF1A1 | -3.842391 |
| LOC101927926 | -3.816989 |
| IQSEC2 | -3.812483 |
| CYP26C1 | -3.7802284 |
| ANKRD20A2 | -3.7586548 |
| EYS | -3.7541404 |
| TMEM238 | -3.7162519 |
| BET1 | -3.6767845 |
| TTC9 | -3.652306 |
| CETP | -3.6430953 |
| ELP2 | -3.6260996 |
| ND4L | -3.6181715 |
| HCG4B | -3.6037686 |
| B4GALNT3 | -3.5839815 |
| SSBP1 | -3.58157 |
| ZNF526 | -3.5573874 |
| KRTAP20-3 | -3.5403597 |
| KCTD9 | -3.537336 |
| HBA2 | -3.5196261 |
| VTCN1 | -3.5136516 |
| RHOA | -3.4953916 |
| LINC00707 | -3.4799683 |
| RPS29 | -3.4788642 |
| SLC35A2 | -3.4770322 |
| STARD9 | -3.445275 |
| UNCX | -3.4235177 |
| RTN4RL2 | -3.4117215 |
| ZNF805 | -3.3977175 |
| GOLGA7B | -3.3810558 |
| FKBP11 | -3.349005 |
| PPIP5K1 | -3.3407178 |
| PXYLP1 | -3.340702 |
| DNM1 | -3.3357394 |
| GALNT12 | -3.3328896 |
| RPL23AP64 | -3.3007326 |
| CLDN2 | -3.2669644 |
| ADAMTSL1 | -3.2604141 |
| RPL23A | -3.244974 |
| PDE4A | -3.2425168 |
| TXNRD1 | -3.239731 |
| ZNF789 | -3.1826544 |
| RPS27 | -3.1789815 |
| WSCD2 | -3.1663604 |
| LMAN2 | -3.1607544 |
| ZDHHC20 | -3.1470294 |
| PCDHGA8 | -3.1466885 |
| TBX6 | -3.1425025 |
| GPX1 | -3.0975118 |
| RPL28 | -3.0726528 |
| MICU2 | -3.0679302 |
| TNFSF10 | -3.051285 |
| HES2 | -3.047139 |
| CRYBB3 | -3.0418372 |
| LHX5 | -3.041695 |
| MROH2A | -3.0397594 |
| HSFY2 | -3.0277689 |
| RPS13 | -3.0215907 |
| IVD | -3.020596 |
| LAMA5 | -3.0190392 |
| IFT43 | -2.9888468 |
| TNF | -2.9710038 |
| LOC440117 | -2.9678636 |
| GPALPP1 | -2.964373 |
| WDR55 | -2.9533925 |
| VKORC1L1 | -2.9422767 |
| POR | -2.9235296 |
| STK39 | -2.922162 |
| LOC101927450 | -2.9112666 |
| LCE1D | -2.9084756 |
| UQCC3 | -2.908414 |
| PSMG2 | -2.9071798 |
| SLFN12L | -2.8984625 |
| LFNG | -2.892031 |
| LOC284600 | -2.8914375 |
| XLOC_l2_015885 | -2.886974 |
| PHC1 | -2.8823998 |
| lnc-TP53TG3-7 | -2.8791013 |
| ALX3 | -2.872987 |
| RPS6KA5 | -2.8514693 |
| TNRC18 | -2.8431013 |
| TTYH1 | -2.834943 |
| ENKUR | -2.8331501 |
| ADAT2 | -2.828926 |
| LINC00890 | -2.8164325 |
| CHMP1A | -2.812927 |
| NUPL2 | -2.805299 |
| DZIP1L | -2.7976465 |
| FAM209B | -2.7959719 |
| AMACR | -2.7892282 |
| AP3M2 | -2.7852094 |
| RPS3A | -2.7834394 |
| DPH7 | -2.7817216 |
| CYMP | -2.7597044 |
| SIPA1L1 | -2.7470765 |
| KCNK4 | -2.7430196 |
| ZMYND12 | -2.7184663 |
| TNIP1 | -2.7080743 |
| ANKRD62 | -2.7025943 |
| DST | -2.7010677 |
| LOC100129931 | -2.6945124 |
| LRRC71 | -2.6936128 |
| TPM3 | -2.6880498 |
| ZNF181 | -2.6840425 |
| SETDB2 | -2.6791809 |
| RPS14 | -2.6779008 |
| LINC00944 | -2.6771007 |
| SVEP1 | -2.6565547 |
| DUX4 | -2.6502244 |
| ANKRD11 | -2.6447115 |
| ZNF703 | -2.644261 |
| RPL23A | -2.643595 |
| CPLX2 | -2.6396327 |
| UNC80 | -2.6382334 |
| USF2 | -2.6245425 |
| LCE1C | -2.6230006 |
| FTH1 | -2.62055 |
| NDUFA9 | -2.6080625 |
| RASGRF2-AS1 | -2.608026 |
| TBC1D9 | -2.6035752 |
| GPR52 | -2.6025085 |
| CCDC17 | -2.596115 |
| ARHGEF18 | -2.5914888 |
| COL27A1 | -2.5713975 |
| ADAMTSL5 | -2.570908 |
| RNA5-8S5 | -2.5642006 |
| GOLGA8O | -2.5639565 |
| TRABD | -2.563516 |
| TMEM120B | -2.5559452 |
| CASKIN1 | -2.5419216 |
| ST6GAL2 | -2.4895525 |
| HIST1H2BN | -2.4820776 |
| LOC146513 | -2.4801786 |
| NOL4L | -2.4696784 |
| RETNLB | -2.468453 |
| MAGI1 | -2.457882 |
| AP4S1 | -2.4480932 |
| ZNF865 | -2.4442382 |
| PCDH18 | -2.44266 |
| LINC01006 | -2.4299822 |
| SV2B | -2.4295006 |
| UMAD1 | -2.416581 |
| PTRF | -2.4127805 |
| HIST3H3 | -2.4116757 |
| SNORD114-16 | -2.4040592 |
| IQCF2 | -2.396925 |
| LINC00920 | -2.394663 |
| RNF216 | -2.386869 |
| ASB10 | -2.384546 |
| EPOR | -2.3839407 |
| ZP1 | -2.3832662 |
| HLA-E | -2.3794205 |
| LINC00908 | -2.3705113 |
| KIAA0040 | -2.3650627 |
| RPL19 | -2.365041 |
| C5orf63 | -2.3621645 |
| TGM2 | -2.356687 |
| RALBP1 | -2.3528702 |
| ARHGAP4 | -2.3510542 |
| NPPA-AS1 | -2.329426 |
| LINC00202-1 | -2.3280668 |
| SMG1 | -2.3154588 |
| APOBEC3G | -2.3152165 |
| MAFK | -2.3132846 |
| MGC39584 | -2.3009222 |
| LINC01344 | -2.29777 |
| RPS18 | -2.2961938 |
| CFAP58 | -2.292213 |
| COG4 | -2.2909677 |
| WNT5B | -2.2888124 |
| ZBTB43 | -2.287389 |
| LINC00928 | -2.2819264 |
| CORO1B | -2.2763865 |
| ATG14 | -2.2661638 |
| IGSF6 | -2.2615187 |
| LRRC73 | -2.255854 |
| LOC286178 | -2.2547255 |
| TCHH | -2.2537076 |
| SEMA5B | -2.2457097 |
| PDE9A | -2.2409983 |
| MALAT1 | -2.2387538 |
| GGTLC1 | -2.2366962 |
| MRPS36 | -2.234611 |
| MS4A15 | -2.2281108 |
| KIAA1671 | -2.2275288 |
| BCKDK | -2.2219858 |
| PPIL3 | -2.2167866 |
| H3F3A | -2.215057 |
| RPL26 | -2.2127955 |
| ZBTB7B | -2.203314 |
| C9orf153 | -2.2002282 |
| WDR86-AS1 | -2.1981523 |
| CEACAM16 | -2.1936657 |
| MCF2L | -2.1925392 |
| GUK1 | -2.188168 |
| SPATA45 | -2.183231 |
| RNF126 | -2.18241 |
| HES5 | -2.1809404 |
| RNF6 | -2.17924 |
| SNX21 | -2.1745274 |
| ZNF684 | -2.1731234 |
| SLC3A1 | -2.1718826 |
| ZNF133 | -2.1685426 |
| SUCLG2 | -2.1682878 |
| PHF20 | -2.1655602 |
| ITFG3 | -2.1640348 |
| PMF1 | -2.1579938 |
| INTS6 | -2.1561887 |
| RAET1E | -2.1544971 |
| WNK2 | -2.1537597 |
| RPS16 | -2.149254 |
| RPL38 | -2.1484175 |
| SLC26A8 | -2.1373868 |
| TTTY1 | -2.1336741 |
| FAM215A | -2.1176095 |
| ANXA4 | -2.1136208 |
| NCALD | -2.1100333 |
| CADM1 | -2.0973203 |
| TRIM34 | -2.096537 |
| MIAT | -2.0887313 |
| ZNF777 | -2.0886288 |
| KIAA0753 | -2.0881572 |
| CCDC144A | -2.086464 |
| SCARF2 | -2.0861177 |
| SP8 | -2.0798595 |
| PLEC | -2.077859 |
| SNORD3B-1 | -2.0728638 |
| TPT1 | -2.072854 |
| GSTT2B | -2.0669212 |
| RPL23 | -2.0643802 |
| EDNRB | -2.0626354 |
| MSANTD4 | -2.0626152 |
| DIABLO | -2.0619962 |
| RPL13A | -2.0604768 |
| ACY1 | -2.057881 |
| FHIT | -2.0570621 |
| C20orf144 | -2.0563827 |
| MUTYH | -2.0525284 |
| DNAJB8 | -2.052238 |
| LOC389641 | -2.0465062 |
| ANKS6 | -2.0418093 |
| DLX6 | -2.0410576 |
| HAR1B | -2.0397966 |
| CCDC3 | -2.0382302 |
| RPSAP58 | -2.036128 |
| LINC00963 | -2.0358806 |
| PRKXP1 | -2.035742 |
| HLA-F | -2.0229416 |
| HELZ2 | -2.0209608 |
| ZNF135 | -2.008473 |
| ELF3 | -2.0076706 |
| FAM163B | -2.00505 |
| LINC00602 | -2.004288 |
| CES1P1 | -2.0042565 |
| ARRDC4 | -2.0032642 |
| SLC6A4 | -2.001537 |
| GGTLC1 | -2.0012257 |
| KANSL1 | 2.0012696 |
| CDH4 | 2.0013754 |
| LOC102467147 | 2.001873 |
| ZC3H7A | 2.0019748 |
| NEK11 | 2.0037436 |
| TOMM40 | 2.0053322 |
| IL6ST | 2.005474 |
| PARVB | 2.0061255 |
| GADD45G | 2.0077755 |
| UBE2Q2L | 2.0140753 |
| PRR3 | 2.014621 |
| ACTR1A | 2.0147038 |
| FST | 2.0153883 |
| SIAH1 | 2.016172 |
| KCNS1 | 2.0179877 |
| HIST1H1D | 2.0180826 |
| SDCCAG3 | 2.0182455 |
| ELK1 | 2.0199058 |
| RAI1 | 2.0207283 |
| MOV10L1 | 2.0227318 |
| CRYBB1 | 2.0230365 |
| FHL3 | 2.0233603 |
| MYNN | 2.0248828 |
| LOC101927151 | 2.0255108 |
| CHMP6 | 2.0270765 |
| HRCT1 | 2.028063 |
| THNSL2 | 2.0286062 |
| PLSCR3 | 2.0300562 |
| TBX15 | 2.0306077 |
| NDUFA6-AS1 | 2.0309741 |
| OR10G8 | 2.0350988 |
| UBE2Q2P2 | 2.035898 |
| PCDHA4 | 2.0365994 |
| HIST1H3I | 2.0374746 |
| ASNS | 2.0376618 |
| DDX17 | 2.0389175 |
| C20orf62 | 2.039547 |
| C2orf74 | 2.0432782 |
| ZNF7 | 2.0432782 |
| CAPZA3 | 2.045788 |
| LOC101927914 | 2.047145 |
| LINC01485 | 2.047999 |
| LINC00483 | 2.0533466 |
| PHOX2B | 2.0546894 |
| TTC29 | 2.0639515 |
| DDX59 | 2.0641468 |
| SNHG5 | 2.0647364 |
| MYO15A | 2.070808 |
| PITPNC1 | 2.0725403 |
| NUP85 | 2.0732994 |
| MGAT1 | 2.0738313 |
| NYNRIN | 2.0751233 |
| ZNF382 | 2.079117 |
| LINC01531 | 2.0799394 |
| DUS4L | 2.083385 |
| C1orf141 | 2.0839994 |
| GMPR | 2.0876958 |
| SCLT1 | 2.0891163 |
| LOC100130476 | 2.0917144 |
| TMEM79 | 2.098563 |
| C11orf84 | 2.104844 |
| ERICH5 | 2.1054883 |
| UBE2I | 2.112368 |
| PSMB5 | 2.1182437 |
| IL7 | 2.1182885 |
| CERCAM | 2.1218958 |
| GON4L | 2.124043 |
| PTK6 | 2.13152 |
| B4GALNT2 | 2.1325617 |
| SNCG | 2.1342807 |
| TNNT3 | 2.1400774 |
| INCA1 | 2.140158 |
| CENPC | 2.1493492 |
| PFDN2 | 2.1542766 |
| ARMCX4 | 2.1591153 |
| HMGCL | 2.1598046 |
| AMT | 2.164849 |
| MIR99AHG | 2.1729913 |
| C19orf73 | 2.1734624 |
| ARL4D | 2.1741436 |
| LOC101927151 | 2.1744323 |
| MRPL10 | 2.1771839 |
| COL4A1 | 2.178625 |
| C4orf22 | 2.1854172 |
| FCGR2A | 2.186514 |
| INO80 | 2.1917002 |
| CCL27 | 2.1972578 |
| CDK11B | 2.2001703 |
| ROBO3 | 2.206767 |
| CLEC10A | 2.2073214 |
| ZNF571-AS1 | 2.2090352 |
| TXNDC5 | 2.2123306 |
| SNX16 | 2.214685 |
| CYP1A1 | 2.222675 |
| ZGRF1 | 2.2272801 |
| CBLN2 | 2.2291636 |
| PRRC2B | 2.233679 |
| DNM1 | 2.2339199 |
| CLHC1 | 2.2372775 |
| PSMD5 | 2.237443 |
| TEKT4P2 | 2.2603707 |
| CENPW | 2.2604306 |
| BHMT2 | 2.2656605 |
| ELP5 | 2.275186 |
| DDN | 2.2828982 |
| LINC00313 | 2.28746 |
| MAP3K13 | 2.3151662 |
| LINC01550 | 2.3192394 |
| SNORD83B | 2.3225963 |
| VWA2 | 2.3359318 |
| C12orf60 | 2.345199 |
| TMEM14A | 2.3594756 |
| ARPC4-TTLL3 | 2.3718343 |
| GIGYF2 | 2.3886602 |
| PTGDS | 2.3918157 |
| ST3GAL1 | 2.3969107 |
| SPRY3 | 2.412305 |
| GRIN2A | 2.4356575 |
| STRC | 2.4638228 |
| ZNF438 | 2.4923222 |
| LUC7L3 | 2.4985907 |
| SAP30BP | 2.505027 |
| LOC100128108 | 2.5324156 |
| BCHE | 2.5330267 |
| SENP8 | 2.5647564 |
| ADM5 | 2.5710628 |
| FKBP9 | 2.5845413 |
| KLHL35 | 2.6595418 |
| FER | 2.6627643 |
| FN3K | 2.6768937 |
| KLHL10 | 2.68338 |
| LINC01585 | 2.6865568 |
| IL13 | 2.7092988 |
| B3GALT5-AS1 | 2.7251916 |
| TFB1M | 2.733027 |
| ARHGEF39 | 2.755798 |
| ARCN1 | 2.7948058 |
| EIF1B-AS1 | 2.8317177 |
| RNF216 | 2.8343003 |
| DHX15 | 2.955164 |
| MRPL36 | 2.9782422 |
| PIN4 | 3.121463 |
| MDM1 | 3.217795 |
| SYT14 | 3.4347878 |
| GMNC | 3.4660532 |
| CCZ1 | 3.476209 |
| TNFAIP2 | 3.5164495 |
| PPAPDC1B | 3.6929998 |
| HCG4B | 3.8014352 |
| PALD1 | 3.9081917 |
| IARS | 4.211243 |
| CPB1 | 4.3320255 |
| LHFP | 4.5610275 |
| IL1RN | 4.617092 |
| IL5 | 4.7715673 |
| ALPK2 | 5.0473113 |
| OSBPL1A | 5.1783714 |
| UBE2U | 5.513724 |
| LOC101927087 | 6.4740906 |
| CYS1 | 7.950653 |
| KIAA1257 | 8.998487 |
| SKA1 | 10.463882 |
| FRAS1 | 10.481335 |
| CDK5RAP3 | 11.539375 |
| MED30 | 12.533558 |
| LOC101927661 | 12.799273 |
| ZNF704 | 13.452419 |
| CSMD1 | 13.581577 |
| TRIM24 | 14.504196 |
| CECR2 | 14.569444 |
| LINC00857 | 15.094208 |
| SEL1L3 | 16.813782 |
| IFNA16 | 26.411564 |
| IFNA10 | 36.546635 |
| AFAP1L2 | 36.832138 |
| LINC00320 | 42.651627 |
| C8orf37 | 42.797047 |
| CCDC14 | 49.687695 |
| IL6R | 60.495083 |
| RPSAP58 | 62.95407 |
| FAM153A | 71.58133 |
| LAIR1 | 80.03919 |
| UBE3D | 133.32567 |
| CCL13 | 133.60953 |
| F13 | 134.06277 |
| RCHY1 | 193.78471 |

**Table S2**. List of differentially expressed genes between normal and epileptic children with significantly enriched for CRE motifs, related with Table S1.

CCR2

ZSWIM7

MYO6

SNAPC2

BMS1

TBX22

MKLN1-AS

EFTUD2

IDH3B

DSP

FOSL1

PRR19

COX6B2

ZNF713

CAMK2B

CDK11A

HTR1F

GPATCH2

BTAF1

AQP7P3

CRYAA

TRIM41

TBL2

VWDE

GOLGA2

OXLD1

ZNF385C

ALOX15

FUT6

RPL21

TTC28

POLR2J2

AS3MT

SNORA15

GPR155

MAST4

RNU4ATAC

NR2F6

ZNF581

CTAGE7P

NMNAT1

CHPF

SLC25A16

CEP70

RN7SL1

FAM74A4

OGFRL1

ZNF816

TMEM191B

TMEM138

MGA

BTN3A1

TRIQK

SBDS

CYP2W1

LINC00608

LINC00399

CBR1

MIR600HG

TRNT1

GREB1

SLC16A12

HIST1H4H

PRTFDC1

MYCNUT

CHAT

NRXN1

CCDC22

PDE4DIP

ATP6V1G3

FAM200B

C14orf1

RPS15

GUCY1B3

LINC01405

NKX3-1

RPL9

HAUS1

HSCB

TMEM8C

PDCD4-AS1

CXADR

RPL13

HLA-G

ZNF593

SLC35E4

HLA-A

TTPAL

RPS11

LINC01001

ALPP

BOC

SMAD3

PIEZO1

FTSJ2

C10orf105

GLUD1P3

RPL21

CASC5

MORN4

PGLYRP1

ACBD7

ADAMTS7

RPL5

EEF1A1

TTC9

CETP

ELP2

B4GALNT3

SSBP1

ZNF526

KCTD9

RHOA

LINC00707

STARD9

UNCX

GOLGA7B

FKBP11

PPIP5K1

DNM1

GALNT12

RPL23AP64

CLDN2

RPL23A

PDE4A

TXNRD1

ZNF789

RPS27

LMAN2

ZDHHC20

PCDHGA8

TBX6

TNFSF10

CRYBB3

RPS13

LAMA5

IFT43

TNF

GPALPP1

VKORC1L1

POR

PSMG2

LFNG

RPS6KA5

TTYH1

ENKUR

DZIP1L

FAM209B

AP3M2

RPS3A

DPH7

CYMP

ZMYND12

TNIP1

ANKRD62

LRRC71

ZNF181

RPS14

LINC00944

SVEP1

ANKRD11

RPL23A

FTH1

NDUFA9

RASGRF2-AS1

TBC1D9

CCDC17

ARHGEF18

ADAMTSL5

GOLGA8O

TMEM120B

ST6GAL2

NOL4L

RETNLB

MAGI1

SV2B

HIST3H3

IQCF2

RNF216

ASB10

EPOR

LINC00908

RPL19

C5orf63

TGM2

ARHGAP4

NPPA-AS1

SMG1

APOBEC3G

MAFK

MGC39584

RPS18

COG4

WNT5B

ZBTB43

LINC00928

CORO1B

ATG14

PDE9A

MRPS36

MS4A15

KIAA1671

H3F3A

RPL26

ZBTB7B

C9orf153

CEACAM16

MCF2L

SPATA45

RNF126

RNF6

SNX21

ZNF684

SLC3A1

SUCLG2

PHF20

RPS16

RPL38

SLC26A8

NCALD

CADM1

TRIM34

MIAT

ZNF777

CCDC144A

SCARF2

SP8

PLEC

TPT1

GSTT2B

RPL23

DIABLO

RPL13A

ACY1

C20orf144

ANKS6

DLX6

CCDC3

RPSAP58

PRKXP1

HLA-F

HELZ2

ELF3

LINC00602

KANSL1

NEK11

TOMM40

IL6ST

PARVB

UBE2Q2L

PRR3

SIAH1

ELK1

MOV10L1

CRYBB1

FHL3

CHMP6

HRCT1

PLSCR3

TBX15

NDUFA6-AS1

OR10G8

PCDHA4

HIST1H3I

ASNS

DDX17

C2orf74

ZNF7

CAPZA3

LINC01485

LINC00483

MYO15A

NUP85

MGAT1

NYNRIN

ZNF382

LINC01531

C1orf141

GMPR

SCLT1

C11orf84

ERICH5

PSMB5

CERCAM

B4GALNT2

TNNT3

INCA1

PFDN2

HMGCL

AMT

C19orf73

ARL4D

MRPL10

COL4A1

C4orf22

INO80

CDK11B

ROBO3

CLEC10A

ZNF571-AS1

CYP1A1

ZGRF1

CBLN2

PRRC2B

DNM1

CENPW

ELP5

MAP3K13

LINC01550

SNORD83B

VWA2

C12orf60

TMEM14A

ARPC4-TTLL3

STRC

ZNF438

LUC7L3

SAP30BP

SENP8

FER

KLHL10

LINC01585

B3GALT5-AS1

TFB1M

ARCN1

RNF216

DHX15

MRPL36

MDM1

CCZ1

TNFAIP2

CPB1

LHFP

IL1RN

IL5

CYS1

SKA1

FRAS1

CDK5RAP3

ZNF704

CSMD1

TRIM24

AFAP1L2

LINC00320

CCDC14

IL6R

RPSAP58

LAIR1

CCL13

RCHY1

**Table S3.** List of differentially expressed genes with CRE motifs in epileptic children, and the effect of VPA administration. The box in color represents the effect of VPA. Orange: Up-regulated, Purple: Down-regulated.

|  | **Effect of VPA at 6 months** | | | | **Effect of VPA at 12 months** | | | |
| --- | --- | --- | --- | --- | --- | --- | --- | --- |
| **Epileptic CREB genes** | **No Change** | **Back Control** | **Under Control** | **Above Control** | **No Change** | **Back Control** | **Under Control** | **Above Control** |
| **UP-REGULATED** |  |  |  |  |  |  |  |  |
| CCR2 |  |  |  |  |  |  |  |  |
| ZSWIM7 |  |  |  |  |  |  |  |  |
| MYO6 |  |  |  |  |  |  |  |  |
| SNAPC2 |  |  |  |  |  |  |  |  |
| BMS1 |  |  |  |  |  |  |  |  |
| TBX22 |  |  |  |  |  |  |  |  |
| MKLN1-AS |  |  |  |  |  |  |  |  |
| EFTUD2 |  |  |  |  |  |  |  |  |
| IDH3B |  |  |  |  |  |  |  |  |
| DSP |  |  |  |  |  |  |  |  |
| FOSL1 |  |  |  |  |  |  |  |  |
| PRR19 |  |  |  |  |  |  |  |  |
| COX6B2 |  |  |  |  |  |  |  |  |
| ZNF713 |  |  |  |  |  |  |  |  |
| CAMK2B |  |  |  |  |  |  |  |  |
| CDK11A |  |  |  |  |  |  |  |  |
| HTR1F |  |  |  |  |  |  |  |  |
| GPATCH2 |  |  |  |  |  |  |  |  |
| BTAF1 |  |  |  |  |  |  |  |  |
| AQP7P3 |  |  |  |  |  |  |  |  |
| CRYAA |  |  |  |  |  |  |  |  |
| TRIM41 |  |  |  |  |  |  |  |  |
| TBL2 |  |  |  |  |  |  |  |  |
| VWDE |  |  |  |  |  |  |  |  |
| GOLGA2 |  |  |  |  |  |  |  |  |
| OXLD1 |  |  |  |  |  |  |  |  |
| ZNF385C |  |  |  |  |  |  |  |  |
| ALOX15 |  |  |  |  |  |  |  |  |
| FUT6 |  |  |  |  |  |  |  |  |
| RPL21 |  |  |  |  |  |  |  |  |
| TTC28 |  |  |  |  |  |  |  |  |
| POLR2J2 |  |  |  |  |  |  |  |  |
| AS3MT |  |  |  |  |  |  |  |  |
| SNORA15 |  |  |  |  |  |  |  |  |
| GPR155 |  |  |  |  |  |  |  |  |
| MAST4 |  |  |  |  |  |  |  |  |
| RNU4ATAC |  |  |  |  |  |  |  |  |
| NR2F6 |  |  |  |  |  |  |  |  |
| ZNF581 |  |  |  |  |  |  |  |  |
| CTAGE7P |  |  |  |  |  |  |  |  |
| NMNAT1 |  |  |  |  |  |  |  |  |
| CHPF |  |  |  |  |  |  |  |  |
| SLC25A16 |  |  |  |  |  |  |  |  |
| CEP70 |  |  |  |  |  |  |  |  |
| RN7SL1 |  |  |  |  |  |  |  |  |
| FAM74A4 |  |  |  |  |  |  |  |  |
| OGFRL1 |  |  |  |  |  |  |  |  |
| ZNF816 |  |  |  |  |  |  |  |  |
| TMEM191B |  |  |  |  |  |  |  |  |
| TMEM138 |  |  |  |  |  |  |  |  |
| MGA |  |  |  |  |  |  |  |  |
| BTN3A1 |  |  |  |  |  |  |  |  |
| TRIQK |  |  |  |  |  |  |  |  |
| SBDS |  |  |  |  |  |  |  |  |
| CYP2W1 |  |  |  |  |  |  |  |  |
| LINC00608 |  |  |  |  |  |  |  |  |
| LINC00399 |  |  |  |  |  |  |  |  |
| CBR1 |  |  |  |  |  |  |  |  |
| MIR600HG |  |  |  |  |  |  |  |  |
| TRNT1 |  |  |  |  |  |  |  |  |
| GREB1 |  |  |  |  |  |  |  |  |
| SLC16A12 |  |  |  |  |  |  |  |  |
| HIST1H4H |  |  |  |  |  |  |  |  |
| PRTFDC1 |  |  |  |  |  |  |  |  |
| MYCNUT |  |  |  |  |  |  |  |  |
| CHAT |  |  |  |  |  |  |  |  |
| NRXN1 |  |  |  |  |  |  |  |  |
| CCDC22 |  |  |  |  |  |  |  |  |
| PDE4DIP |  |  |  |  |  |  |  |  |
| ATP6V1G3 |  |  |  |  |  |  |  |  |
| FAM200B |  |  |  |  |  |  |  |  |
| C14orf1 |  |  |  |  |  |  |  |  |
| RPS15 |  |  |  |  |  |  |  |  |
| GUCY1B3 |  |  |  |  |  |  |  |  |
| LINC01405 |  |  |  |  |  |  |  |  |
| NKX3-1 |  |  |  |  |  |  |  |  |
| RPL9 |  |  |  |  |  |  |  |  |
| HAUS1 |  |  |  |  |  |  |  |  |
| HSCB |  |  |  |  |  |  |  |  |
| TMEM8C |  |  |  |  |  |  |  |  |
| PDCD4-AS1 |  |  |  |  |  |  |  |  |
| CXADR |  |  |  |  |  |  |  |  |
| RPL13 |  |  |  |  |  |  |  |  |
| HLA-G |  |  |  |  |  |  |  |  |
| ZNF593 |  |  |  |  |  |  |  |  |
| SLC35E4 |  |  |  |  |  |  |  |  |
| HLA-A |  |  |  |  |  |  |  |  |
| TTPAL |  |  |  |  |  |  |  |  |
| RPS11 |  |  |  |  |  |  |  |  |
| LINC01001 |  |  |  |  |  |  |  |  |
| ALPP |  |  |  |  |  |  |  |  |
| BOC |  |  |  |  |  |  |  |  |
| SMAD3 |  |  |  |  |  |  |  |  |
| PIEZO1 |  |  |  |  |  |  |  |  |
| FTSJ2 |  |  |  |  |  |  |  |  |
| C10orf105 |  |  |  |  |  |  |  |  |
| GLUD1P3 |  |  |  |  |  |  |  |  |
| RPL21 |  |  |  |  |  |  |  |  |
| CASC5 |  |  |  |  |  |  |  |  |
| MORN4 |  |  |  |  |  |  |  |  |
| PGLYRP1 |  |  |  |  |  |  |  |  |
| ACBD7 |  |  |  |  |  |  |  |  |
| ADAMTS7 |  |  |  |  |  |  |  |  |
| RPL5 |  |  |  |  |  |  |  |  |
| EEF1A1 |  |  |  |  |  |  |  |  |
| TTC9 |  |  |  |  |  |  |  |  |
| CETP |  |  |  |  |  |  |  |  |
| ELP2 |  |  |  |  |  |  |  |  |
| B4GALNT3 |  |  |  |  |  |  |  |  |
| SSBP1 |  |  |  |  |  |  |  |  |
| ZNF526 |  |  |  |  |  |  |  |  |
| KCTD9 |  |  |  |  |  |  |  |  |
| RHOA |  |  |  |  |  |  |  |  |
| LINC00707 |  |  |  |  |  |  |  |  |
| STARD9 |  |  |  |  |  |  |  |  |
| UNCX |  |  |  |  |  |  |  |  |
| GOLGA7B |  |  |  |  |  |  |  |  |
| FKBP11 |  |  |  |  |  |  |  |  |
| PPIP5K1 |  |  |  |  |  |  |  |  |
| DNM1 |  |  |  |  |  |  |  |  |
| GALNT12 |  |  |  |  |  |  |  |  |
| RPL23AP64 |  |  |  |  |  |  |  |  |
| CLDN2 |  |  |  |  |  |  |  |  |
| RPL23A |  |  |  |  |  |  |  |  |
| PDE4A |  |  |  |  |  |  |  |  |
| TXNRD1 |  |  |  |  |  |  |  |  |
| ZNF789 |  |  |  |  |  |  |  |  |
| RPS27 |  |  |  |  |  |  |  |  |
| LMAN2 |  |  |  |  |  |  |  |  |
| ZDHHC20 |  |  |  |  |  |  |  |  |
| PCDHGA8 |  |  |  |  |  |  |  |  |
| TBX6 |  |  |  |  |  |  |  |  |
| TNFSF10 |  |  |  |  |  |  |  |  |
| CRYBB3 |  |  |  |  |  |  |  |  |
| RPS13 |  |  |  |  |  |  |  |  |
| LAMA5 |  |  |  |  |  |  |  |  |
| IFT43 |  |  |  |  |  |  |  |  |
| TNF |  |  |  |  |  |  |  |  |
| GPALPP1 |  |  |  |  |  |  |  |  |
| VKORC1L1 |  |  |  |  |  |  |  |  |
| POR |  |  |  |  |  |  |  |  |
| PSMG2 |  |  |  |  |  |  |  |  |
| LFNG |  |  |  |  |  |  |  |  |
| RPS6KA5 |  |  |  |  |  |  |  |  |
| TTYH1 |  |  |  |  |  |  |  |  |
| ENKUR |  |  |  |  |  |  |  |  |
| DZIP1L |  |  |  |  |  |  |  |  |
| FAM209B |  |  |  |  |  |  |  |  |
| AP3M2 |  |  |  |  |  |  |  |  |
| RPS3A |  |  |  |  |  |  |  |  |
| DPH7 |  |  |  |  |  |  |  |  |
| CYMP |  |  |  |  |  |  |  |  |
| ZMYND12 |  |  |  |  |  |  |  |  |
| TNIP1 |  |  |  |  |  |  |  |  |
| ANKRD62 |  |  |  |  |  |  |  |  |
| LRRC71 |  |  |  |  |  |  |  |  |
| ZNF181 |  |  |  |  |  |  |  |  |
| RPS14 |  |  |  |  |  |  |  |  |
| LINC00944 |  |  |  |  |  |  |  |  |
| SVEP1 |  |  |  |  |  |  |  |  |
| ANKRD11 |  |  |  |  |  |  |  |  |
| RPL23A |  |  |  |  |  |  |  |  |
| FTH1 |  |  |  |  |  |  |  |  |
| NDUFA9 |  |  |  |  |  |  |  |  |
| RASGRF2-AS1 |  |  |  |  |  |  |  |  |
| TBC1D9 |  |  |  |  |  |  |  |  |
| CCDC17 |  |  |  |  |  |  |  |  |
| ARHGEF18 |  |  |  |  |  |  |  |  |
| ADAMTSL5 |  |  |  |  |  |  |  |  |
| GOLGA8O |  |  |  |  |  |  |  |  |
| TMEM120B |  |  |  |  |  |  |  |  |
| ST6GAL2 |  |  |  |  |  |  |  |  |
| NOL4L |  |  |  |  |  |  |  |  |
| RETNLB |  |  |  |  |  |  |  |  |
| MAGI1 |  |  |  |  |  |  |  |  |
| SV2B |  |  |  |  |  |  |  |  |
| HIST3H3 |  |  |  |  |  |  |  |  |
| IQCF2 |  |  |  |  |  |  |  |  |
| RNF216 |  |  |  |  |  |  |  |  |
| ASB10 |  |  |  |  |  |  |  |  |
| EPOR |  |  |  |  |  |  |  |  |
| LINC00908 |  |  |  |  |  |  |  |  |
| RPL19 |  |  |  |  |  |  |  |  |
| C5orf63 |  |  |  |  |  |  |  |  |
| TGM2 |  |  |  |  |  |  |  |  |
| ARHGAP4 |  |  |  |  |  |  |  |  |
| NPPA-AS1 |  |  |  |  |  |  |  |  |
| SMG1 |  |  |  |  |  |  |  |  |
| APOBEC3G |  |  |  |  |  |  |  |  |
| MAFK |  |  |  |  |  |  |  |  |
| MGC39584 |  |  |  |  |  |  |  |  |
| RPS18 |  |  |  |  |  |  |  |  |
| COG4 |  |  |  |  |  |  |  |  |
| WNT5B |  |  |  |  |  |  |  |  |
| ZBTB43 |  |  |  |  |  |  |  |  |
| LINC00928 |  |  |  |  |  |  |  |  |
| CORO1B |  |  |  |  |  |  |  |  |
| ATG14 |  |  |  |  |  |  |  |  |
| PDE9A |  |  |  |  |  |  |  |  |
| MRPS36 |  |  |  |  |  |  |  |  |
| MS4A15 |  |  |  |  |  |  |  |  |
| KIAA1671 |  |  |  |  |  |  |  |  |
| H3F3A |  |  |  |  |  |  |  |  |
| RPL26 |  |  |  |  |  |  |  |  |
| ZBTB7B |  |  |  |  |  |  |  |  |
| C9orf153 |  |  |  |  |  |  |  |  |
| CEACAM16 |  |  |  |  |  |  |  |  |
| MCF2L |  |  |  |  |  |  |  |  |
| SPATA45 |  |  |  |  |  |  |  |  |
| RNF126 |  |  |  |  |  |  |  |  |
| RNF6 |  |  |  |  |  |  |  |  |
| SNX21 |  |  |  |  |  |  |  |  |
| ZNF684 |  |  |  |  |  |  |  |  |
| SLC3A1 |  |  |  |  |  |  |  |  |
| SUCLG2 |  |  |  |  |  |  |  |  |
| PHF20 |  |  |  |  |  |  |  |  |
| RPS16 |  |  |  |  |  |  |  |  |
| RPL38 |  |  |  |  |  |  |  |  |
| SLC26A8 |  |  |  |  |  |  |  |  |
| NCALD |  |  |  |  |  |  |  |  |
| CADM1 |  |  |  |  |  |  |  |  |
| TRIM34 |  |  |  |  |  |  |  |  |
| MIAT |  |  |  |  |  |  |  |  |
| ZNF777 |  |  |  |  |  |  |  |  |
| CCDC144A |  |  |  |  |  |  |  |  |
| SCARF2 |  |  |  |  |  |  |  |  |
| SP8 |  |  |  |  |  |  |  |  |
| PLEC |  |  |  |  |  |  |  |  |
| TPT1 |  |  |  |  |  |  |  |  |
| GSTT2B |  |  |  |  |  |  |  |  |
| RPL23 |  |  |  |  |  |  |  |  |
| DIABLO |  |  |  |  |  |  |  |  |
| RPL13A |  |  |  |  |  |  |  |  |
| ACY1 |  |  |  |  |  |  |  |  |
| C20orf144 |  |  |  |  |  |  |  |  |
| ANKS6 |  |  |  |  |  |  |  |  |
| DLX6 |  |  |  |  |  |  |  |  |
| CCDC3 |  |  |  |  |  |  |  |  |
| RPSAP58 |  |  |  |  |  |  |  |  |
| PRKXP1 |  |  |  |  |  |  |  |  |
| HLA-F |  |  |  |  |  |  |  |  |
| HELZ2 |  |  |  |  |  |  |  |  |
| ELF3 |  |  |  |  |  |  |  |  |
| LINC00602 |  |  |  |  |  |  |  |  |
|  |  |  |  |  |  |  |  |  |
| **DOWN-REGULATED** |  |  |  |  |  |  |  |  |
| KANSL1 |  |  |  |  |  |  |  |  |
| NEK11 |  |  |  |  |  |  |  |  |
| TOMM40 |  |  |  |  |  |  |  |  |
| IL6ST |  |  |  |  |  |  |  |  |
| PARVB |  |  |  |  |  |  |  |  |
| UBE2Q2L |  |  |  |  |  |  |  |  |
| PRR3 |  |  |  |  |  |  |  |  |
| SIAH1 |  |  |  |  |  |  |  |  |
| ELK1 |  |  |  |  |  |  |  |  |
| MOV10L1 |  |  |  |  |  |  |  |  |
| CRYBB1 |  |  |  |  |  |  |  |  |
| FHL3 |  |  |  |  |  |  |  |  |
| CHMP6 |  |  |  |  |  |  |  |  |
| HRCT1 |  |  |  |  |  |  |  |  |
| PLSCR3 |  |  |  |  |  |  |  |  |
| TBX15 |  |  |  |  |  |  |  |  |
| NDUFA6-AS1 |  |  |  |  |  |  |  |  |
| OR10G8 |  |  |  |  |  |  |  |  |
| PCDHA4 |  |  |  |  |  |  |  |  |
| HIST1H3I |  |  |  |  |  |  |  |  |
| ASNS |  |  |  |  |  |  |  |  |
| DDX17 |  |  |  |  |  |  |  |  |
| C2orf74 |  |  |  |  |  |  |  |  |
| ZNF7 |  |  |  |  |  |  |  |  |
| CAPZA3 |  |  |  |  |  |  |  |  |
| LINC01485 |  |  |  |  |  |  |  |  |
| LINC00483 |  |  |  |  |  |  |  |  |
| MYO15A |  |  |  |  |  |  |  |  |
| NUP85 |  |  |  |  |  |  |  |  |
| MGAT1 |  |  |  |  |  |  |  |  |
| NYNRIN |  |  |  |  |  |  |  |  |
| ZNF382 |  |  |  |  |  |  |  |  |
| LINC01531 |  |  |  |  |  |  |  |  |
| C1orf141 |  |  |  |  |  |  |  |  |
| GMPR |  |  |  |  |  |  |  |  |
| SCLT1 |  |  |  |  |  |  |  |  |
| C11orf84 |  |  |  |  |  |  |  |  |
| ERICH5 |  |  |  |  |  |  |  |  |
| PSMB5 |  |  |  |  |  |  |  |  |
| CERCAM |  |  |  |  |  |  |  |  |
| B4GALNT2 |  |  |  |  |  |  |  |  |
| TNNT3 |  |  |  |  |  |  |  |  |
| INCA1 |  |  |  |  |  |  |  |  |
| PFDN2 |  |  |  |  |  |  |  |  |
| HMGCL |  |  |  |  |  |  |  |  |
| AMT |  |  |  |  |  |  |  |  |
| C19orf73 |  |  |  |  |  |  |  |  |
| ARL4D |  |  |  |  |  |  |  |  |
| MRPL10 |  |  |  |  |  |  |  |  |
| COL4A1 |  |  |  |  |  |  |  |  |
| C4orf22 |  |  |  |  |  |  |  |  |
| INO80 |  |  |  |  |  |  |  |  |
| CDK11B |  |  |  |  |  |  |  |  |
| ROBO3 |  |  |  |  |  |  |  |  |
| CLEC10A |  |  |  |  |  |  |  |  |
| ZNF571-AS1 |  |  |  |  |  |  |  |  |
| CYP1A1 |  |  |  |  |  |  |  |  |
| ZGRF1 |  |  |  |  |  |  |  |  |
| CBLN2 |  |  |  |  |  |  |  |  |
| PRRC2B |  |  |  |  |  |  |  |  |
| DNM1 |  |  |  |  |  |  |  |  |
| CENPW |  |  |  |  |  |  |  |  |
| ELP5 |  |  |  |  |  |  |  |  |
| MAP3K13 |  |  |  |  |  |  |  |  |
| LINC01550 |  |  |  |  |  |  |  |  |
| SNORD83B |  |  |  |  |  |  |  |  |
| VWA2 |  |  |  |  |  |  |  |  |
| C12orf60 |  |  |  |  |  |  |  |  |
| TMEM14A |  |  |  |  |  |  |  |  |
| ARPC4-TTLL3 |  |  |  |  |  |  |  |  |
| STRC |  |  |  |  |  |  |  |  |
| ZNF438 |  |  |  |  |  |  |  |  |
| LUC7L3 |  |  |  |  |  |  |  |  |
| SAP30BP |  |  |  |  |  |  |  |  |
| SENP8 |  |  |  |  |  |  |  |  |
| FER |  |  |  |  |  |  |  |  |
| KLHL10 |  |  |  |  |  |  |  |  |
| LINC01585 |  |  |  |  |  |  |  |  |
| B3GALT5-AS1 |  |  |  |  |  |  |  |  |
| TFB1M |  |  |  |  |  |  |  |  |
| ARCN1 |  |  |  |  |  |  |  |  |
| RNF216 |  |  |  |  |  |  |  |  |
| DHX15 |  |  |  |  |  |  |  |  |
| MRPL36 |  |  |  |  |  |  |  |  |
| MDM1 |  |  |  |  |  |  |  |  |
| CCZ1 |  |  |  |  |  |  |  |  |
| TNFAIP2 |  |  |  |  |  |  |  |  |
| CPB1 |  |  |  |  |  |  |  |  |
| LHFP |  |  |  |  |  |  |  |  |
| IL1RN |  |  |  |  |  |  |  |  |
| IL5 |  |  |  |  |  |  |  |  |
| CYS1 |  |  |  |  |  |  |  |  |
| SKA1 |  |  |  |  |  |  |  |  |
| FRAS1 |  |  |  |  |  |  |  |  |
| CDK5RAP3 |  |  |  |  |  |  |  |  |
| ZNF704 |  |  |  |  |  |  |  |  |
| CSMD1 |  |  |  |  |  |  |  |  |
| TRIM24 |  |  |  |  |  |  |  |  |
| AFAP1L2 |  |  |  |  |  |  |  |  |
| LINC00320 |  |  |  |  |  |  |  |  |
| CCDC14 |  |  |  |  |  |  |  |  |
| IL6R |  |  |  |  |  |  |  |  |
| RPSAP58 |  |  |  |  |  |  |  |  |
| LAIR1 |  |  |  |  |  |  |  |  |
| CCL13 |  |  |  |  |  |  |  |  |
| RCHY1 |  |  |  |  |  |  |  |  |
